# A Null Model for Pearson Coexpression Networks

**DOI:** 10.1371/journal.pone.0128115

**Published:** 2015-06-01

**Authors:** Andrea Gobbi, Giuseppe Jurman

**Affiliations:** Fondazione Bruno Kessler, Trento, Italy; University of Jaén, SPAIN

## Abstract

Gene coexpression networks inferred by correlation from high-throughput profiling such as microarray data represent simple but effective structures for discovering and interpreting linear gene relationships. In recent years, several approaches have been proposed to tackle the problem of deciding when the resulting correlation values are statistically significant. This is most crucial when the number of samples is small, yielding a non-negligible chance that even high correlation values are due to random effects. Here we introduce a novel hard thresholding solution based on the assumption that a coexpression network inferred by randomly generated data is expected to be empty. The threshold is theoretically derived by means of an analytic approach and, as a deterministic independent null model, it depends only on the dimensions of the starting data matrix, with assumptions on the skewness of the data distribution compatible with the structure of gene expression levels data. We show, on synthetic and array datasets, that the proposed threshold is effective in eliminating all false positive links, with an offsetting cost in terms of false negative detected edges.

## Introduction

Universally acknowledged by the scientific community as the basic task of systems biology, network inference is the prototypical procedure for moving from the classical reductionist approach to the novel paradigm of data-driven complex systems in the interpretation of biological processes [1].

The goal of all network inference (or network reconstruction) procedures is the detection of the topology (*i.e.*, the wiring diagram) of a graph whose nodes belong to a given set of biological entities, starting from measurements of the entities themselves. In the last fifteen years, the reconstruction of the regulation mechanism of a gene network and of the interactions among proteins from high-throughput data such as expression microarray or, more recently, from Next Generation Sequencing data has become a major line of research for laboratories worldwide. The proposed approaches rely on techniques ranging from deterministic to stochastic, and their number is constantly growing. Nonetheless, network inference is still considered an open, unsolved problem [2]. In fact, in many practical cases, the performance of the reconstruction algorithms are poor, due to several factors limiting inference accuracy [3, 4] to the point of making it no better than a coin toss in some situations [5]. The major problem is the under-determinacy of the task [6], due to the overwhelming number of interactions to be predicted starting from a (usually) small number of available measurements. In general, size and quality of available data are critical factors for all inference algorithms.

In what follows the impact of data size is discussed for one of the simplest inference techniques, *i.e.*, the gene coexpression network, where interaction strength between two genes is a function of the correlation between the corresponding expression levels across the available tissue samples. Despite its simplicity, the number of studies in the literature based on coexpression networks is still a large fraction of all manuscripts in systems biology [7–13], including Next Generation Sequencing data [14]. The underlying biological hypothesis is that functionally related genes have similar expression patterns [15], and thus that coexpression is correlated with functional relationships, although this does not imply causality. This implies for instance that genes that are closer in a biological network are likely to have more similar expression [16, 17]: this hypothesis has been validated to some extent, for example by Jensen *et al.* in [18]. However, a caveat is mandatory here: since correlation is a univariate method, it is unable to capture the relations occurring among genes when the independence hypothesis does not hold, which is a common situation in the -omics datasets [19, 20]. To overcome this problem, characterized by small correlation values between functionally related genes, multivariate approaches are required [21], also in the network case [22].

Notwithstanding this limitation, as highlighted in [23], correlation can help unveil the underlying cellular processes, since coordinated coexpression of genes encode interacting proteins. The Pearson Correlation Coefficient (PCC for short) is used as the standard measure, although alternative correlation measures can be also employed: see for instance [24] for a comparative review of eight well known association metrics. Furthermore, coexpression analysis has been intensively used as an effective algorithm to explore the system-level functionality of genes, sometimes outperforming much more refined approaches [25, 26]. The observation that simpler approaches such as correlation can be superior even on synthetic data has been explained by some authors [27, 28] with the difficulties of a complex algorithm in detecting the subtleties of combinatorial regulation. Coexpression networks can also capture more important features than the conventional differential expression approach [29], and their use has been extended to other tasks, for instance the investigation of complex biological traits [30]. Finally, these networks can be crucial for understanding regulatory mechanisms [31], for the development of personalised medicine [32] or, more recently, in metagenomics [33].

However, as noted in [34], correlation between genes may sometimes be due to unobserved factors affecting expression levels. Moreover, deciding when a given correlation value between two nodes can be deemed statistically significant and thus worthwhile for assigning a link connecting them is a major issue affecting coexpression networks [35]. This translates mathematically into choosing (a function of) a suitable threshold, as in the case of mutual information and relevance networks [36]. As reported in [37], in the literature statistical methods for testing the correlations are underdeveloped, and thresholding is often overlooked even in important studies [38]. The two main approaches known in the literature to solve this problem can be classified as soft or hard thresholding. Soft thresholding is adopted in a well-known framework called Weighted Gene Coexpression Network Analysis (WGCNA) [39], recently used also for other network types [40, 41]. All genes are mutually connected, and the weight of a link is a positive power of the absolute value of PCC, where the exponent is chosen as the best fit of the resulting network according to a scale-free model [42, 43]. This approach, without discarding any correlations, promotes high correlation values and penalises low values. In the hard thresholding approach, instead, only correlation values larger than the threshold are taken into account, and an unweighted link is set for each of these values, so that a binary network is generated (see [44] for one of the earliest references). Clearly, an incorrectly chosen threshold value can jeopardise the obtained results with false negative links (for too strict a threshold) or false positive links (for too loose a threshold). Many heuristics have been proposed for setting the threshold values, such as using the False Discovery Rate [45–47], using a cross-validation strategy [48], the *p*-value of the correlation test [15, 32], employing partial correlation [49, 50], using rank-based techniques [51–53], more complex randomisation techniques [54], or keeping only values exceeding a minimum acceptable strength (MAS) level specified by the threshold [55]. Alternative approaches have been recently studied, evaluating the correlation distribution, both experimentally [56] and theoretically [57]. However, these studies focus on the level of single interaction rather than considering the whole network.

Moreover, in many studies in the literature, the threshold is not chosen according to an algorithmic procedure, but referring to standard choices [58–61], or to heuristics not directly related to the correlation values, but rather with the resulting network topology [62–71]. In [72] a comparison of some coexpression thresholds is shown on a few microarray datasets. Furthermore, each threshold choice yields a compromise between detecting artefacts (false positives) and neglecting existing connections (false negatives) [56], which can be driven by the cost attached to the task studied. For instance, the optimized threshold selection procedure in [73] attains a very low false positive rate by using a permutation-based strategy [74].

Here we propose a new a priori model for the computation of a hard threshold based on the assumption that a random coexpression graph should not have any edges. This threshold follows from the work of Fisher [75] and Bevington [76], and, as a deterministic independent null model, it depends only on the dimensions of the starting data matrix, with assumptions on the skewness of the data distribution compatible with the structure of gene expression levels data [77, 78]. Hence, the procedure to obtain the threshold is not stochastic and thus deeply different from approaches based on permutation tests [74]. Further, this model is non-parametric, because its only input is the data themselves and no additional quantity needs to be tuned. By definition, this threshold is aimed at minimising the possible false positive links, paying a price in terms of false negative detected edges. This characterising property makes this method especially useful when the intrinsic cost function associated with the studied task is biased towards penalising false positives. We conclude by demonstrating the procedure first on a synthetic dataset and then on an ovarian epithelial carcinoma dataset on a large cohort of 285 cases [79, 80].

## A Motivating Example

We show hereafter an example of a common situation arising in the small sample size setting, where PCC can reach extremely high values possibly leading to the detection of false positives.

Consider the HepatoCellular Carcinoma (HCC) dataset, first introduced in [81] and later used in [82] and publicly available at the Gene Expression Omnibus (GEO) http://www.ncbi.nlm.nih.gov/geo with accession number GSE6857. The dataset collects 482 tissue samples from 241 patients affected by HCC. For each patient, a sample from cancerous hepatic tissue and a sample from surrounding non-cancerous hepatic tissue were extracted, hybridised on the Ohio State University CCC MicroRNA Microarray Version 2.0 platform consisting of 11,520 probes collecting expressions of 250 non-redundant human and 200 mouse miRNA. After a preprocessing phase including imputation of missing values [83] and discarding probes corresponding to non-human (mouse and controls) miRNA, the resulting dataset HCC includes 240 + 240 paired samples described by 210 human miRNA, with the cohort consisting of 210 male and 30 female patients. In particular, consider now the three microRNA identified as hsa.mir.010b.precNo1, hsa.mir.016a.chr13 and hsa.mir.016b.chr3. A search by sequence with the miRBase web-service [84] shows that the two probes hsa.mir.016a.chr13, and hsa.mir.016b.chr3 share a high alignment score (*e*-value: 5 · 10^−4^[85]), while no link due to coherent coexpression is known between each of these probes and hsa.mir.010b.precNo1. Consistent with this observation, the absolute PCC |*ρ*| on the dataset H consisting of the 210 tumoral samples from male patients is

$$\begin{array}{ccc} |\rho_{H}\left(\text{hsa}.\text{mir}.016\text{a}.\text{chr}13,\text{hsa}.\text{mir}.016\text{b}.\text{chr}3\right)| & = & 0.969\\ |\rho_{H}\left(\text{hsa}.\text{mir}.010\text{b}.\text{precNo}1,\text{hsa}.\text{mir}.016\text{b}.\text{chr}3\right)| & = & 0.536. \end{array}$$

When we restrict the analysis to a suitable small subset of patients, different situations can occur. Consider in fact the following sub-cohort of 10 patients

$$\begin{array}{ccc} S & = & \{03-457,02-354,03-146,03-467,03-037,\\ & & 03-033,03-280,03-205,02-421,02-432\}\;. \end{array}$$

**Fig 1:**
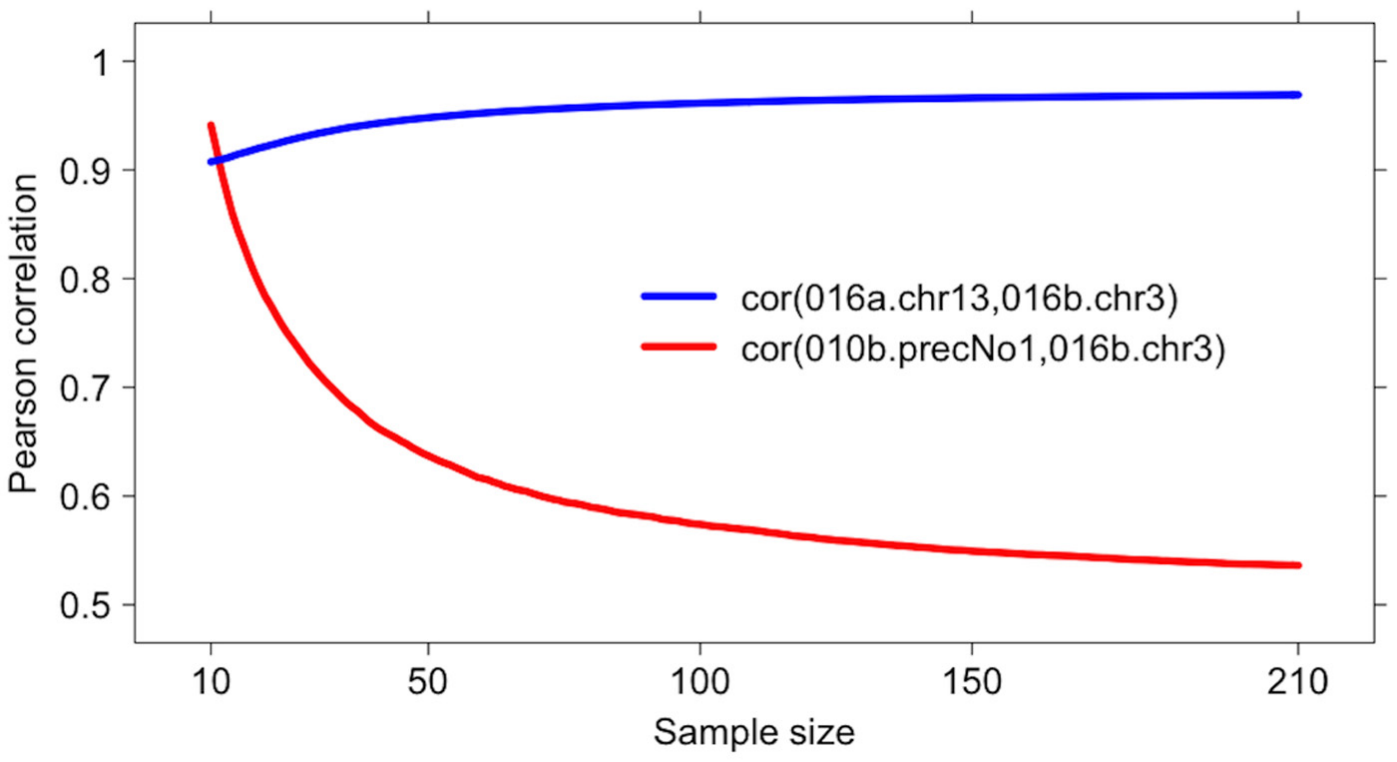
HCC dataset: PCC versus sample size for the coexpression of hsa.mir.016a.chr13 with hsa.mir.016b.chr3 (blue line) and with hsa.mir.010b.precNo1 (red line). When the correlation is computed on the 10-sample set S the correlation between the two uncoexpressed probes is even higher than the correlation between the two probes sharing an almost identical alignment. When more samples are randomly added, the blue line slightly increases, while the red line quickly drops to the final value 0.536 on the whole dataset H. Both curves are averaged over 1000 randomisations of the added samples, keeping the sub-cohort S constant.

On S, PCC reads as follows:

$$\begin{array}{ccc} |\rho_{S}\left(\text{hsa}.\text{mir}.016\text{a}.\text{chr}13,\text{hsa}.\text{mir}.016\text{b}.\text{chr}3\right)| & = & 0.907\\ |\rho_{S}\left(\text{hsa}.\text{mir}.010\text{b}.\text{precNo}1,\text{hsa}.\text{mir}.016\text{b}.\text{chr}3\right)| & = & 0.941, \end{array}$$

that is, the correlation between the two unrelated miRNA on S is very high and even higher than the correlation of the two aligned probes. The curves (averaged on 1000 runs) of PCC versus the sample size when the remaining samples are increasingly and randomly added (while S remains the same across the 1000 runs) are shown in Fig 1. When sample size increases, |*ρ*(hsa.mir.010b.precNo1,hsa.mir.016b.chr3)| quickly drops down to 0.536, while the correlation of the two aligned probes remains almost constant.

Clearly, if the task were to build a PCC coexpression network *N*_S_ on S, whatever the chosen hard threshold *t*, the network *N*_S_ would include either a false positive or a false negative link (or even both, for 0.907 ≤ *t* ≤ 0.941). Note that the probability of extracting 10-sample sub-cohorts S′ satisfying

$$\begin{array}{ccc} & & |\rho_{S^{\prime }}\left(\text{hsa}.\text{mir}.010\text{b}.\text{precNo}1,\text{hsa}.\text{mir}.016\text{b}.\text{chr}3\right)|>\\ & & \;|\rho_{S^{\prime }}\left(\text{hsa}.\text{mir}.016\text{a}.\text{chr}13,\text{hsa}.\text{mir}.016\text{b}.\text{chr}3\right)|>0.9 \end{array}$$

is about 0.03%, but when the constraints are relaxed the probability of detecting artificially high correlation values |*ρ*_S′_(hsa.mir.010b.precNo1,hsa.mir.016b.chr3)| > 0.75 raises to about 6.2% for this dataset. Situations like those shown in this example regularly occur when building coexpression networks, supporting the need for an algorithmic solution to the problem of hard thresholding in the small sample size setup.

## Methods

### Distribution of PCC

Given a real number 0 < *p* < 1, define the function

$$\begin{array}{c} F(n,p)=P(|\rho (x,y)|>p)\;, \end{array}$$ (1)

where *x* and *y* are two independent normal vectors of length *n* and *ρ* is the PCC.

The results in [75, 86–92] and the symmetry of *ρ* yield that *F*(*n*, *p*) has the following close form:

$$\begin{array}{c} F\left(n,p\right)=P\left(|\rho \left(x,y\right)|>p\right)=\frac{2}{\sqrt{\pi }}\frac{\Gamma (\frac{n-1}{2})}{\Gamma (\frac{n-2}{2})}\int_{0}^{\arccos p}\sin^{n-3}\left(\vartheta \right)d\vartheta \;, \end{array}$$ (2)

where Γ(*x*) is the Gamma function $\Gamma (x)=\int_{0}^{+\infty }t^{x-1}e^{-t}\text{d}t$. In Text A in S1 Text we also propose a novel proof of Eq (2) by means of an analytic argument stemming from the work of Bevington in [76, Ch. 7]. Moreover, Eq (2) is also a good approximation in the case of a non-normal distribution of *x* and *y* when data skewness can be bounded [93], because of the generalization shown in [89, 94–96]. Non-Gaussian asymmetric distributions can occasionally be detected in some array studies [78]: however, techniques for reducing the skewness are routinely applied during preprocessing [77], and thus the aforementioned results can be safely used in the microarray framework.

### Coexpression network and threshold selection

The results derived in the previous section are used here to construct a null model for the correlation network, thus yielding a threshold for the inference of a coexpression network from the nodes’ data. As mentioned in the Introduction, these correlation networks are subject to the hypothesis of independence between genes, so they are not detecting higher-order relations for which a multivariate method is needed. In Text E in S1 Text we show a few synthetic and -omics examples of relations among genes that are not captured by coexpression networks.

In the general situation, we are measuring the signal (expression) of *m* probes (genes), in *n* different instances/conditions (samples), to infer the corresponding correlation networks on *m* nodes.

Formally, let $𝒳={\left\{x_{i}\right\}}_{i=1}^{m}$ be a set such that **x**_*i*_ ∈ ℝ^*n*^ ∀*i* = 1, …, *m*. Then the coexpression *p*-graph 𝒢_*p*_ = {*V*, *E*_*p*_} is the graph where

$$\begin{array}{c} V=\left\{v_{1},\ldots ,v_{m}\right\}\;\text{and}\;\left(v_{i},v_{j}\right)\in E_{p}\Leftrightarrow \left|\rho \left(x_{i},x_{j}\right)\right|>p\;. \end{array}$$

The first result characterizes the coexpression graphs in terms of null models:

**Proposition 1** If the vectors **x**_*i*_ are sampled from the uniform distribution, the graph 𝒢_*p*_ is an Erdös-Rényi model [97] with *m* nodes and probability *F*(*n*, *p*) as in Eq (2).

The proof follows immediately from the definition of 𝒢_*p*_ and Eq (2).

**Definition** Using the results in the previous section, the *secure threshold* $\bar{p}$ is defined as follows, for an arbitrarily small *ɛ* > 0:

$$\begin{array}{c} \bar{p}=\min_{p\in (0,1]}\{F\left(n,p\right)\frac{m(m-1)}{2}\leq 1-\varepsilon \}\;, \end{array}$$ (3)

for *m* nodes measured on *n* samples. As a consequence of [98], stating that the median of the binomial distribution is the integer part of the mean when *F*(*n*, *p*) < 1 − log 2, the secure threshold $\bar{p}$ is the minimum value of *p* such that the corresponding random coexpression network is on average an empty graph *E*_*m*_, *i.e.*, $P({𝒢}_{\bar{p}}=E_{m})\geq \frac{1}{2}$.

**Fig 2:**
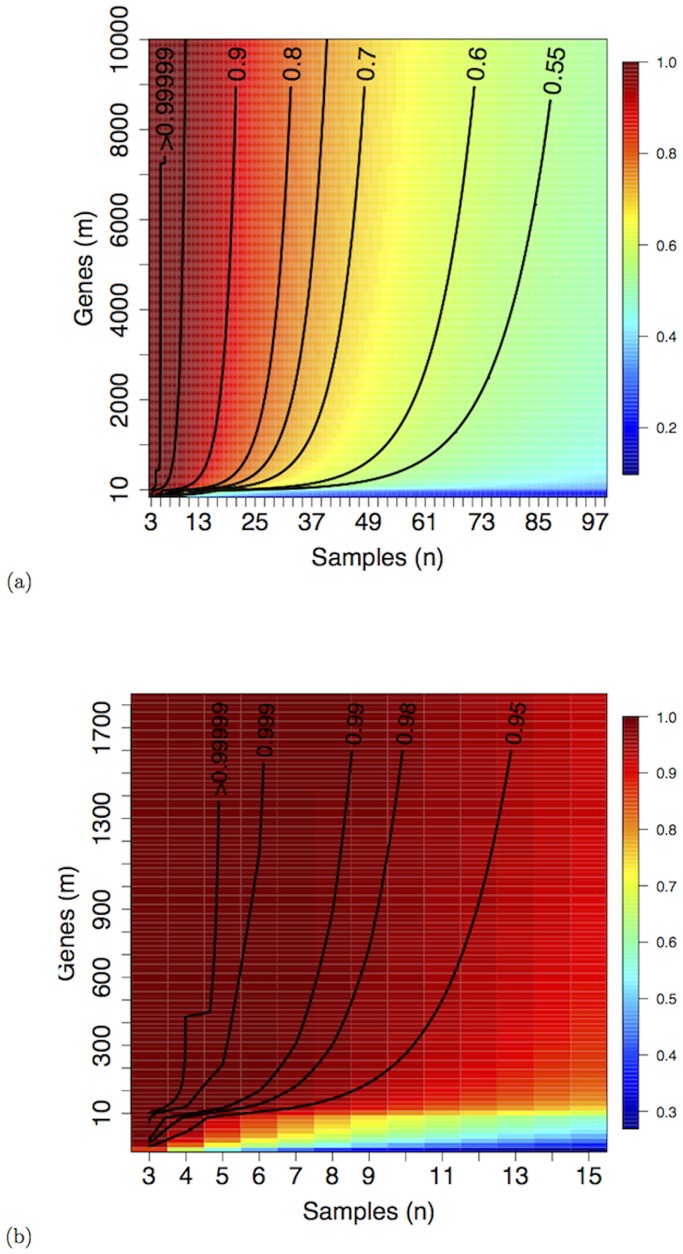
Contour plot of the function $\bar{p}(n,m)$ in the Samples × Genes space on (a) a wide (*n*, *m*) range and (b) zoomed on the small sample size area.

The underlying hypothesis for Eq (3) is the assumption that in a random dataset no edge is expected, since no relation should occur between nodes. Due to its definition, the secure threshold $\bar{p}$ is biased towards avoiding the false positive links, paying a price in terms of false negatives. In Table 1 a collection of values of $\bar{p}$ is listed for different *m* and *n*, while in Fig 2 the contour plot of the function $\bar{p}(n,m)$ is shown first on a large range of values and then zooming on the small sample size area.

**Table 1:** A subset of values of the secure threshold $\bar{p}$ for different number of samples *n* and genes *m*.

m	100	500	1000	2000	10000	50000	100000
n							
8	0.95629	0.98520	0.99070	0.99415	0.99800	0.99932	0.99957
15	0.81681	0.89170	0.91323	0.93036	0.95800	0.97456	0.97949
20	0.73825	0.82388	0.85077	0.87330	0.91286	0.93973	0.94852
30	0.62814	0.71776	0.74817	0.77485	0.82534	0.86367	0.87729
50	0.50225	0.58534	0.61513	0.64213	0.69607	0.74036	0.75705
75	0.41647	0.49026	0.51740	0.54238	0.59353	0.63709	0.65394
100	0.36343	0.42999	0.45477	0.47774	0.52537	0.56662	0.58279

**Fig 3:**
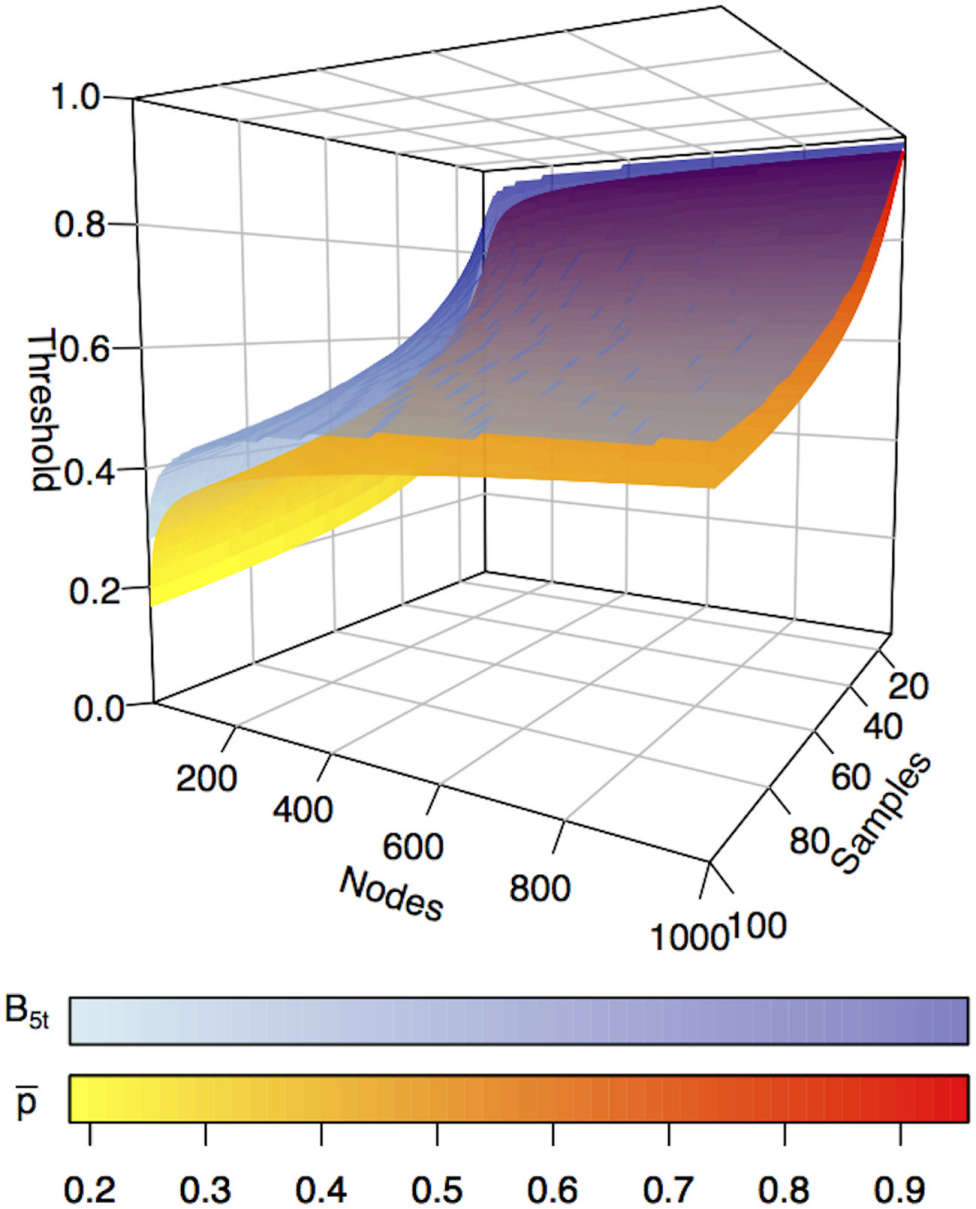
Comparison of the threshold functions $\bar{p}(n,m)$ (yellow-red gradient) and *B*_5, *t*_ (blue gradient) in the Samples × Genes space; darker colors correspond to larger threshold values. The relation $\bar{p}<B_{5,t}$ consistently holds.

As a first example, we compare the secure threshold $\bar{p}$ with the threshold derived by applying the Bonferroni correction procedure [99, 100] to the *p*-value of PCC. Let now Φ_*F*_ and Φ_*t*, *n* − 2_ be the cumulative distribution functions for the Fisher transformation and the Student’s t-distribution with *n* − 2 degrees of freedom, respectively. For a given PCC *ρ* computed between vectors in ℝ^*n*^, let

$$\begin{array}{c} {FT}_{n}\left(\rho \right)=2\Phi_{F}(-|\mathrm{arctanh}\left(\rho \right)|\sqrt{n-3})\;{tT}_{n}\left(\rho \right)=2\Phi_{t,n-2}(-\left|\rho \right|\sqrt{\frac{n-2}{1-r^{2}}}) \end{array}$$ (4)

be the functions computing the *p*-value associated to *ρ* following either the Fisher approximation *FT*_*n*_ [75, 89, 101] or the Student’s t-distribution *tT*_*n*_ [102–106]. Set now the global familywise error rate to 5% significance level (FWER < 0.05): for a network with *m* nodes, by Bonferroni correction, this yields a *p*-value at most $p_{m}=\frac{0.1}{n(n-1)}$ for each link in the graph. By using the inverse functions $B_{5,F}={FT}_{n}^{-1}(p_{m})$ and $B_{5,t}={tT}_{n}^{-1}(p_{m})$ we obtain the corresponding thresholds (Fisher and Student’s t, respectively) attaining the desired significance level. In Table 2 the values of *B*_5, *F*_ and *B*_5, *t*_ are reported for a selection of pairs (*n*, *m*), together with the corresponding secure threshold $\bar{p}$, while in Fig 3 the surface plot of $\bar{p}$ is compared to *B*_5, *t*_.

**Table 2:** The secure threshold and the Bonferroni correction: comparison for different number of samples *n* and genes *m* among the secure threshold $\bar{p}$ and two Bonferroni-derived thresholds *B*_5, *F*_ and *B*_5, *t*_.

m	thr.	5	10	25	50	100	1000
n							
10	$\bar{p}$	0.55	0.71	0.82	0.88	0.92	0.97
	*B*_5, *F*_	0.79	0.85	0.90	0.92	0.94	0.97
	*B*_5, *t*_	0.81	0.87	0.92	0.95	0.97	0.99
15	$\bar{p}$	0.44	0.58	0.70	0.77	0.82	0.91
	*B*_5, *F*_	0.67	0.74	0.80	0.83	0.86	0.92
	*B*_5, *t*_	0.69	0.76	0.83	0.86	0.89	0.95
20	$\bar{p}$	0.38	0.51	0.62	0.69	0.74	0.85
	*B*_5, *F*_	0.60	0.66	0.73	0.76	0.79	0.86
	*B*_5, *t*_	0.61	0.68	0.75	0.79	0.82	0.90
25	$\bar{p}$	0.34	0.46	0.56	0.63	0.68	0.80
	*B*_5, *F*_	0.54	0.61	0.67	0.71	0.74	0.82
	*B*_5, *t*_	0.55	0.62	0.69	0.73	0.77	0.85
50	$\bar{p}$	0.24	0.32	0.41	0.46	0.50	0.62
	*B*_5, *F*_	0.39	0.45	0.50	0.54	0.57	0.66
	*B*_5, *t*_	0.40	0.45	0.51	0.55	0.59	0.68
100	$\bar{p}$	0.17	0.23	0.29	0.33	0.36	0.45
	*B*_5, *F*_	0.28	0.32	0.37	0.40	0.43	0.50
	*B*_5, *t*_	0.28	0.33	0.37	0.40	0.43	0.51

The thresholds *B*_5, *F*_ and *B*_5, *t*_ are computed by applying the Bonferroni correction to the *p*-value of PCC as derived by the Fisher approximation (*B*_5, *F*_) or by the Student’s t-distribution (*B*_5, *t*_), with FWER ≤ 0.05. For each combination of samples *n* and genes *m*, the ranking $\bar{p}<B_{5,F}<B_{5,t}$ consistently holds, and the gaps are narrowing with growing *n* and *m*.

In all cases, the relation $\bar{p}<B_{5,F}<B_{5,t}$ holds, and when *n* < 10 both *B*_5, *F*_ and *B*_5, *t*_ cannot be defined, since the 5% significance level is not reached by any threshold. Both these results do not come unexpected, since the Bonferroni correction is known to be a very conservative procedure, especially when the number of tests is large (see for instance [107–109]) because it ignores existing relations between measurements. This results in a large False Negative Rate. Thus, in many situations in general and in -omics studies in particular, the Bonferroni correction should be substituted by more refined multiple testing approaches [110].

We show now in Table 3 the comparison on a set of synthetic and array datasets of the secure threshold $\bar{p}$ with other well known hard thresholding methods, the clustering coefficient-based threshold C* [67] and the statistical thresholds based on the adjusted *p*-values of 0.01, 0.05 or 0.1, while in Table 4 we compare the secure threshold $\bar{p}$ with the optimal threshold *O* [73] on a collection of array datasets on brain tissues. The threshold *O* is optimized for each dataset (*i.e.*, sample size, data quality and biological structure), considering also the effects induced by the influence of subpopulations on network generation, and comparing to permutated data. Note that for a given pair (*n*, *m*), the threshold $\bar{p}$ is constant, while the threshold *O* depends on the particular dataset. In almost all cases, the threshold $\bar{p}$ is the strictest.

**Table 3:** Comparison of the secure threshold $\bar{p}$ with the clustering coefficient-based threshold C* [67] and the statistical thresholds based on the adjusted p-values B0.01, B0.05 or B0.1 on a collection of synthetic and array datasets.

Dataset type	n	m	C*	B0.01	B0.05	B0.1	$\bar{p}$
Simulated	50	1000	0.57	0.58	0.54	0.52	0.6152
Simulated	25	1000	0.69	0.76	0.72	0.70	0.7956
H-U133P	23	897	0.72	0.78	0.74	0.72	0.8125
H-U133P	10	897	0.78	0.96	0.94	0.93	0.9723
H-U133P	10	675	0.77	0.96	0.93	0.92	0.9681
H-U133P	9	897	0.79	0.97	0.96	0.95	0.9821
H-U133P	8	897	0.81	0.98	0.97	0.96	0.98999
H-U133P	7	897	0.81	0.99	0.99	0.98	0.99558
H-U133P	6	897	0.86	>0.99	>0.99	0.99	0.99872
H-U133P	5	897	0.92	>0.99	>0.99	>0.99	0.99984
H-U133P	4	897	0.99	>0.99	>0.99	>0.99	> 0.9999
H-U133A	4	675	0.99	>0.99	>0.99	>0.99	> 0.9999
H-I6	4	675	0.99	>0.99	>0.99	>0.99	> 0.9999
M-U74	4	401	0.97	>0.99	>0.99	>0.99	0.9999

**Table 4:** Comparison of the secure threshold $\bar{p}$ with the optimal threshold *O* [73] on a collection of array datasets on brain tissues. Note that for a given pair (*n*, *m*), the threshold *p* is constant, while the threshold *O* depends on the particular dataset.

Dataset type	n	m	*O*	$\bar{p}$
H-U133P	30	22277	0.82	0.84570
H-U133P	28	26199	0.85	0.86492
H-U133P	58	29211	0.8	0.68841
H-U133P	56	29211	0.8	0.69741
H-U95av2	50	12453	0.8	0.70261
Agilent	39	12235	0.82	0.76591
H-U133A	44	22383	0.85	0.75203
H-U95av2	47	12453	0.79	0.71857
H-U95av2	46	12453	0.84	0.72411
H-U95av2	50	12453	0.82	0.70261
H-U95av2	59	12453	0.79	0.66011
MOE 430_2	24	25859	0.83	0.89684
MOE 430_2	24	25859	0.78	0.89684
MOE 430_2	24	25859	0.87	0.89684

As shown in the previous section, for a not very skewed distribution, the good approximation provided by the exact formula for *F*(*n*, *p*) given in Eq (2) guarantees the effectiveness of the secure threshold $\bar{p}$ in detecting actual links between nodes. Nonetheless, whenever a stricter threshold is needed, it is still possible to follow the construction proposed, with the following refinement: the edge-creation process in the Erdös-Rényi model follows a binomial distribution, where *n* is the number of trials and *p* the probability associated with the success of a trial. The mean *np* of this distribution is one of the contributing terms in the definition Eq (3) of the secure threshold. To further restrict the number of falsely detected links, the variance term (*np*(1 − *p*) for the binomial distribution) can be added to the original formula through Chebyshev’s inequality:

$$\begin{array}{c} P\left(|X-\mu |\geq k\sigma \right)\leq \frac{1}{k^{2}}\;, \end{array}$$

where *μ* and *σ* are the mean and the standard deviation of *X*. Thus, the definition of secure threshold can be sharpened to ${\tilde{p}}_{k}$ as follows, for an arbitrarily small *ɛ* > 0:

$$\begin{array}{c} {\tilde{p}}_{k}=\min_{p\in (0,1]}\{F\left(n,p\right)\frac{m(m-1)}{2}+k\sqrt{\left(1-F\left(n,p\right)\right)F\left(n,p\right)\frac{m(m-1)}{2}}\leq 1-\varepsilon \}\;. \end{array}$$

For instance, the binomial distribution, for large values of *n*, can be approximated as a normal distribution for which 95.45% of the values lie in the interval (*μ* − 2*σ*, *μ* + 2*σ*). Moreover, the Chebyshev’s inequality implies that at least 96% of the values lie in the interval (*μ* − 5*σ*, *μ* + 5*σ*). Finally, in Text D of S1 Text we show the analogue of Table 1 for ${\tilde{p}}_{2}$ and ${\tilde{p}}_{5}$, respectively.

## Results and Discussion

To conclude, we show the application of the secure threshold $\bar{p}$ in two datasets—synthetic and array—demonstrating its behaviour as a function of data subsampling.

### Synthetic dataset

**Fig 4:**
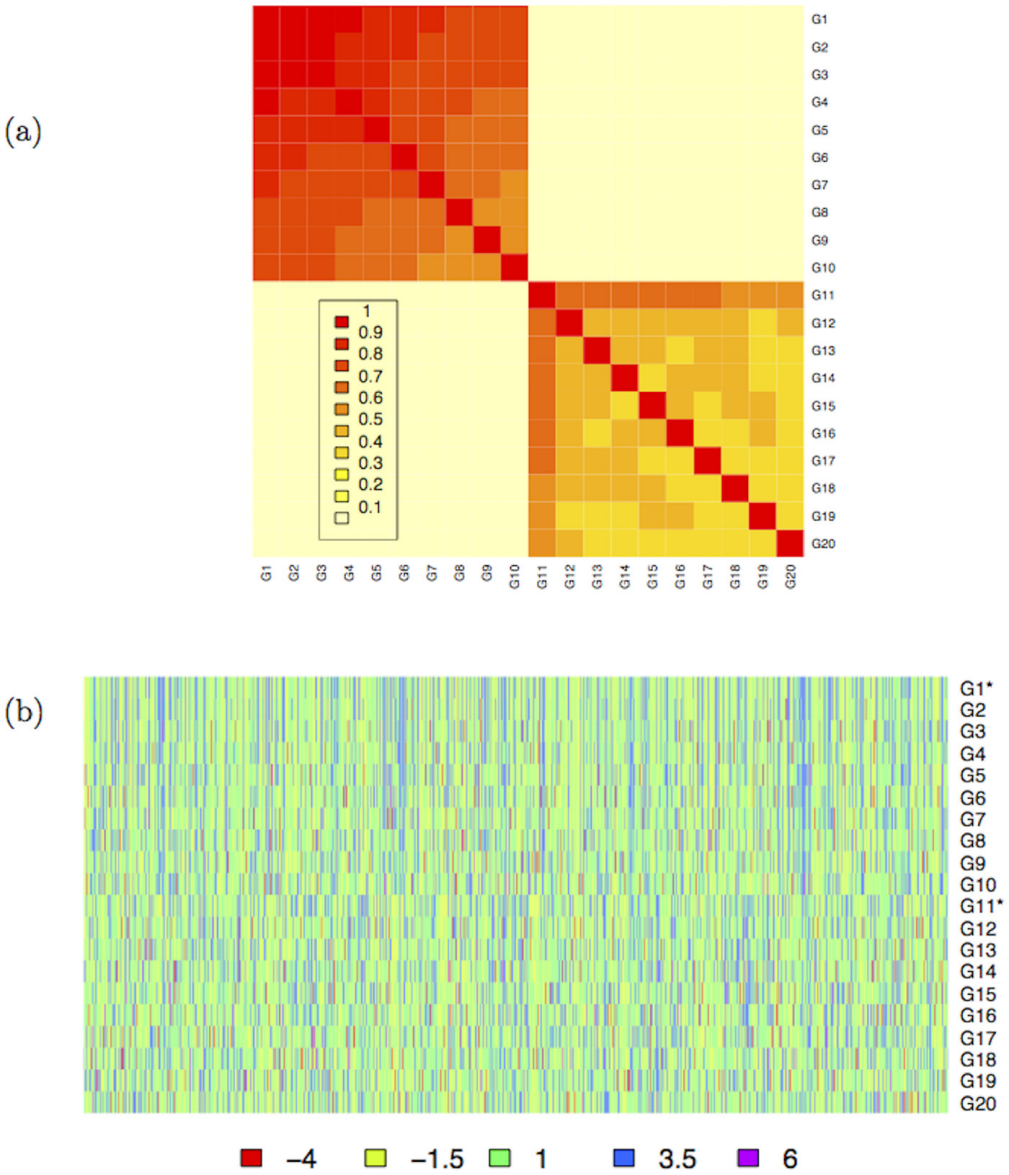
Synthetic dataset 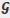: level plot of the structure of the correlation matrix 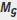 (a) and heatmap of the dataset 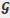 (b). The generating gene expression vectors $G_{1}^{1000}$ and $G_{11}^{1000}$ are marked with *.

In order to construct a simulated microarray dataset 𝒢, we first created a correlation matrix *M*_𝒢_ on 20 genes *G*_1_, …*G*_20_, together with a dataset 𝒢 of the corresponding expression $G_{i}^{1000}$ across 1000 synthetic samples, so that $M_{𝒢}(i,j)=|\rho (G_{i}^{1000},G_{j}^{1000})|$ is the absolute PCC between the expression of the genes *G*_*i*_ and *G*_*j*_ from 𝒢. In particular, *M*_𝒢_ has two 10 × 10 blocks highly correlated on the main diagonal, and two 10 × 10 poorly correlated blocks on the minor diagonal, as shown in Fig 4(a).

These blocks are derived from the following generating rule, given uncorrelated starting element $G_{1}^{1000}$ and $G_{11}^{1000}$:

$$\begin{array}{c} |\rho \left(G_{k}^{1000},G_{j}^{1000}\right)|\approx \{\begin{array}{cc} 1-0.03j & \text{for}\;k=1,2\leq j\leq 10\\ 0.7-0.015j & \text{for}\;k=11,12\leq j\leq 20 \end{array}\;. \end{array}$$

Outside the two main blocks, all correlation values range between 0.002 and 0.074. In order to use *M*_𝒢_ as the ground truth in what follows, all values outside the two blocks on the diagonal are thresholded to zero (*M*_𝒢_)_*i* 10 + *j*_ = (*M*_𝒢_)_*i* 10 + *j*_ = 0 for 1 ≤ *i*, *j* ≤ 10, while entries in the two main blocks are considered as real numbers when evaluating HIM distance and binarized to one when evaluating True/False Positive/Negative links.

In Fig 4(b) we also show the heatmap of the gene expression dataset 𝒢. Then a subset of *n*_*s*_ samples is selected from the starting 1000, and the corresponding coexpression networks is built, for the 100 hard threshold values 0.01j, for 1 ≤ *j* ≤ 100. The secure threshold for these cases are respectively 0.799, 0.596 and 0.389. This procedure is repeated 500 times for each value *n*_*s*_ = 10,20,50. The same experiment is then repeated adding a 20% and a 40% level of Gaussian noise to the original data: for a given signal *s*, we build *s* + *ɛ* with *ɛ* ∈ 𝒩(0, *α* ⋅ (max(*s*) − min(*s*))) for *α* = 0.2,0.4 respectively.

**Fig 5:**
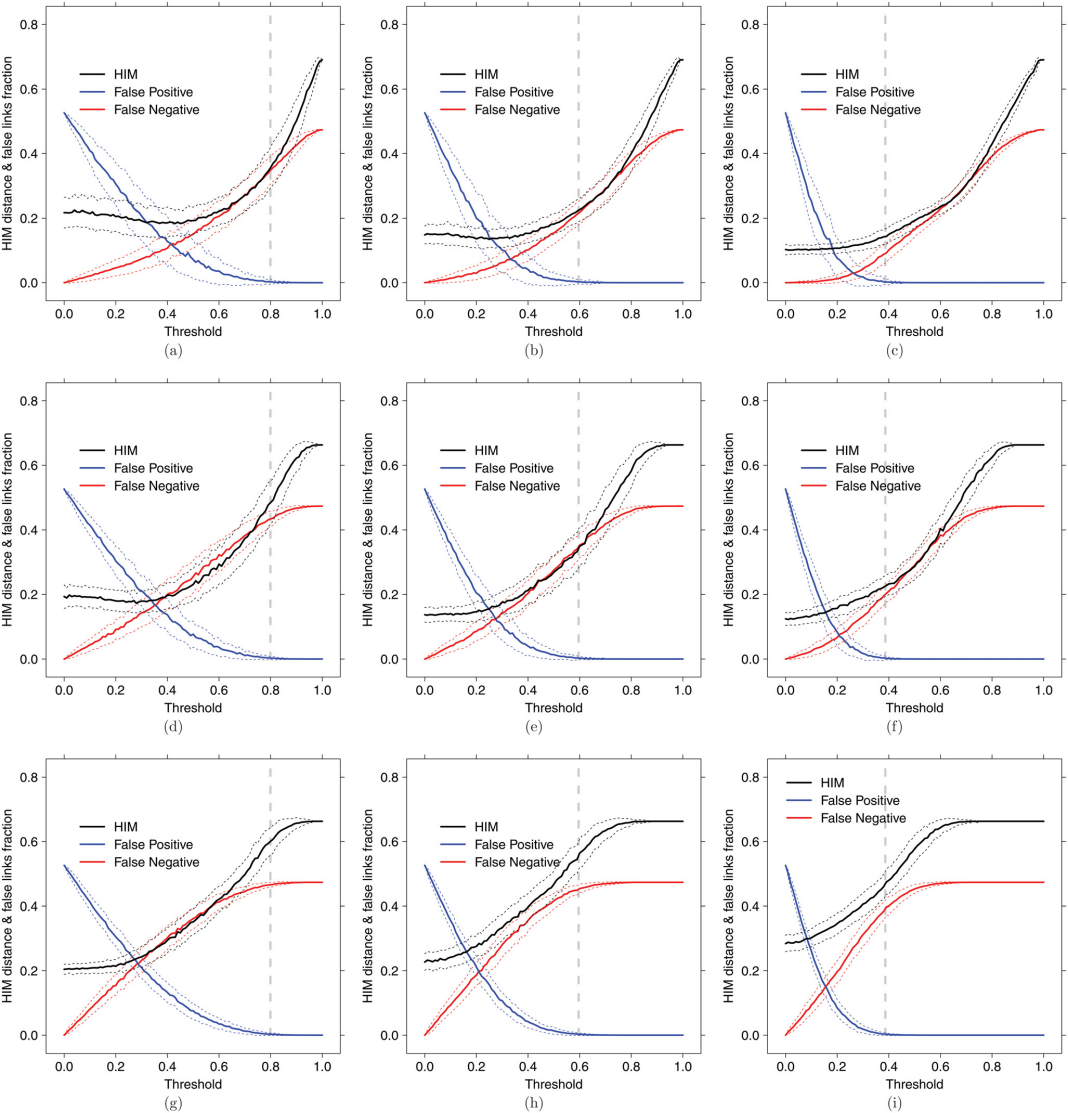
Synthetic dataset 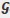. Coexpression inference of the 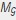 network from random subsampling of the 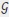 dataset, without noise (a,b,c), with 20% Gaussian noise (d,e,f) and with 40% Gaussian noise (g,h,i), on 10 (a,d,g), 20 (b,e,h) and 50 (c,f,i) samples. Solid lines indicate the mean over 500 replicated instances of HIM distance (black), ratio of False Positive (blue) and ratio of False Negative (red); dotted lines of the same color indicate confidence bars (+/-*σ*), while grey vertical dashed lines correspond to the secure threshold $\bar{p}$.

Using *M*_𝒢_ as the ground truth, for each hard threshold 0.01j we evaluate the ratio of False Positive links (*i.e.*, the quotient between the number of False Positive links and 190, the number of all possible links in a complete undirected network on 20 nodes), the ratio of False Negative links and the Hamming-Ipsen-Mikhailov (HIM) distance from the gold standard. The HIM distance [111, 112] is a metric between networks having the same nodes, ranging from 0 (distance between two identical networks) to 1 (comparison between the complete and the empty graph). The graphs summarizing the experiments are displayed in Fig 5.

In all cases, the secure threshold $\bar{p}$ corresponds to the strictest value yielding a coexpression network with no false positive links included, which is its characterizing property. Moreover, in almost all displayed situations, thresholding at $\bar{p}$ still guarantees an acceptable HIM distance from the ground truth, and a false negative ratio that is always less than 0.4.

### Ovarian cancer

The aforementioned results obtained in the synthetic case are then tested here in a large array study on 285 patients of ovarian cancer at different stages [80], recently used in a comparative study on conservation of coexpressed modules across different pathologies [79]. In detail, the authors profiled the gene expression of 285 predominately high-grade and advanced stage serous cancers of the ovary, fallopian tube, and peritoneum; the samples were hybridized on the Affymetrix Human Genome HG-U133 Plus 2.0 Array, including 54,621 probes. The goal of the original study was to identify novel molecular subtypes of ovarian cancer by gene expression profiling with linkage to clinical and pathologic features. As a major result, the authors presented two ranked gene lists supporting their claim that molecular subtypes show distinct survival characteristics. The two gene lists characterize the Progression Free Survival (PSF) and the Overall Survival (OS) patients, respectively. In each list genes are ranked according to a score weighting their association to target phenotype (PSF or OS): association is stronger for larger absolute values of the score, while the score’s sign is negative for associations with good outcome and positive for associations with poor outcome.

**Fig 6:**
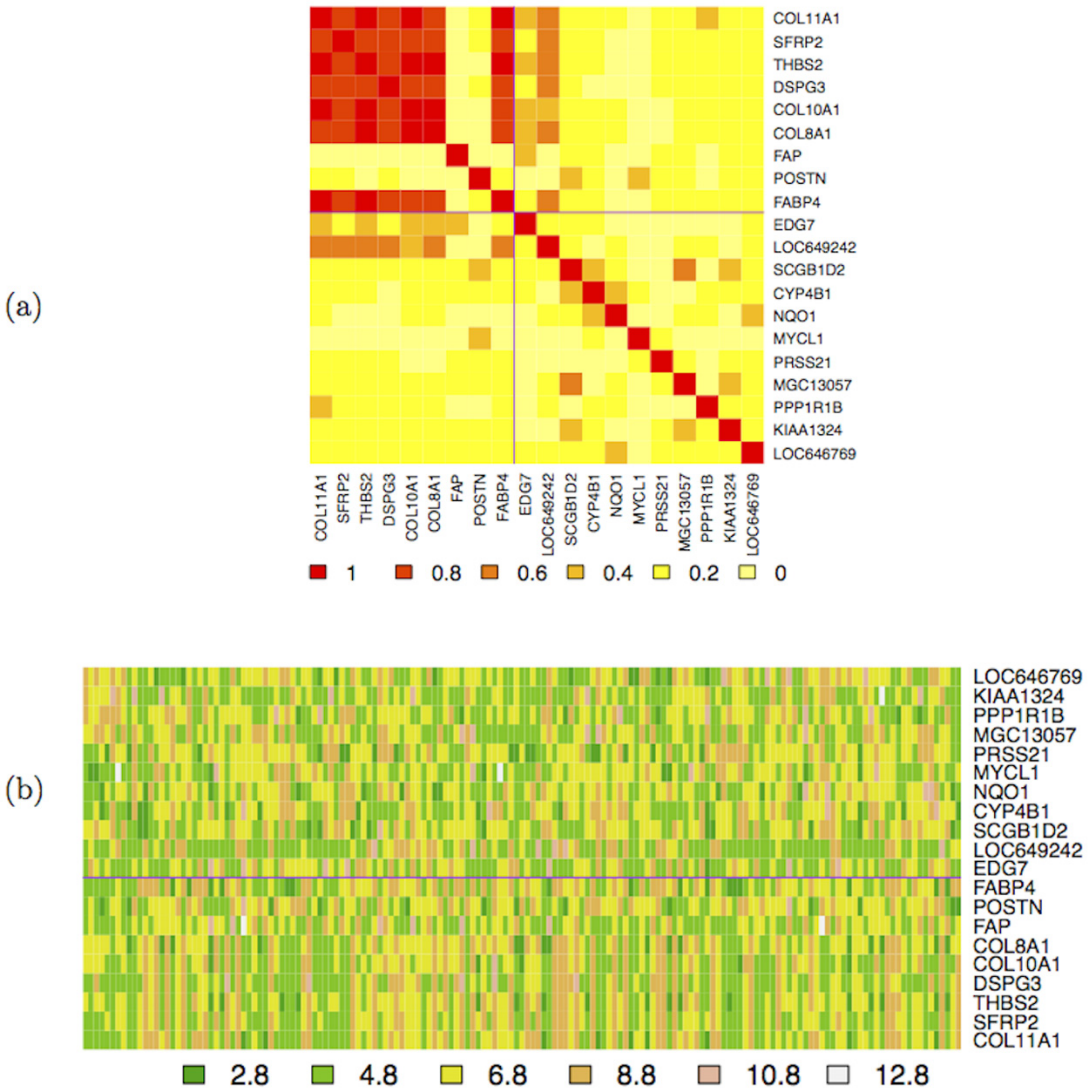
Ovarian cancer dataset 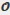. Level plot of the structure of the correlation matrix *O*_*T*_ (a) and heatmap of the Ovarian dataset 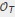 restricted to the set of 20 selected genes *T* (b). Solid lines separate the group of good and poor PFS/OS top genes.

Following the procedure of the previous, synthetic example, first we individuate the sample subset corresponding to the homogeneous cohort of 161 patients with grade three cancer and a set *T* of 20 genes, with 11 genes strongly associated with good PSF or good OS (EDG7, LOC649242, SCGB1D2, CYP4B1, NQO1, MYCL1, PRSS21, MGC13057, PPP1R1B, KIAA1324, LOC646769) and 9 genes strongly associated to poor PFS or poor OS (THBS2, SFRP2, DPSG3, COL11A1, COL10A1, COL8A1, FAP, FABP4, POSTN), thus generating a dataset 𝒪_*T*_ of dimension 161 samples and 20 features. The corresponding absolute PCC matrix *O*_*T*_ is then used as the ground truth for the subsampling experiments, thresholding to zero all values smaller than 0.1: the levelplot of *O*_*T*_ and the heatmap of 𝒪_*T*_ are displayed in Fig 6.

**Fig 7:**
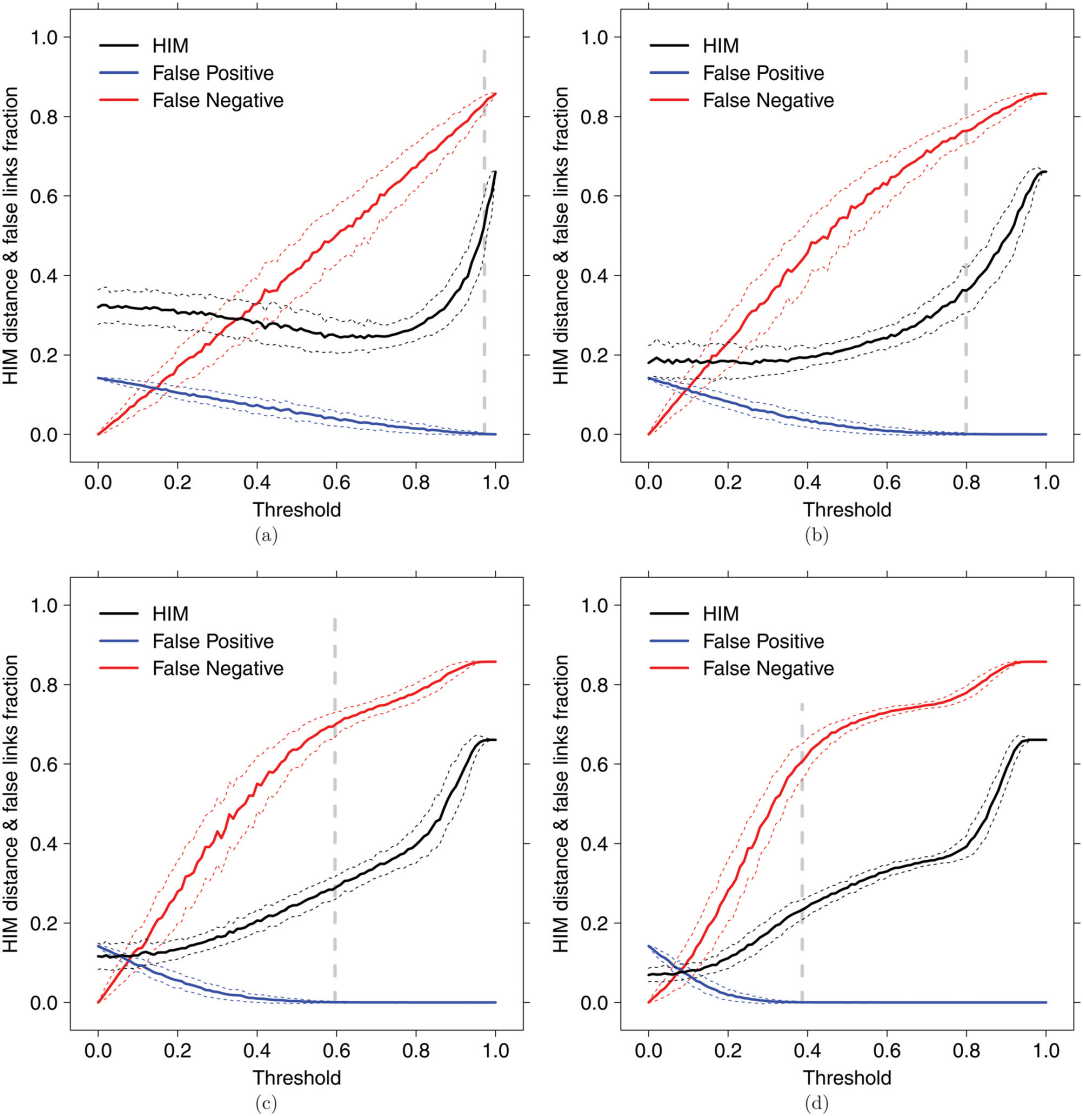
Ovarian cancer dataset 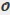. Coexpression inference of the coexpression network from subsampling of 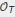, on 5 (a), 10 (b), 20 (c) and 50 (d) samples. Solid lines indicate the mean over 500 replicated instances of HIM distance (black), ratio of False Positive (blue) and ratio of False Negative (red); dotted lines of the same color indicate confidence bars (+/-*σ*), while grey vertical dashed lines correspond to the secure threshold $\bar{p}$.

In these experiments, a random subdataset of *n*_*s*_ samples is extracted from 𝒪_*T*_, and the corresponding absolute PCC coexpression network on the nodes *T* is built, for increasing threshold values. In Fig 7 we report the HIM and the ratio of False Positive and False Negative links for 500 runs of the experiments, separately for *n*_*s*_ = 5,10,20 and 50. Again, the secure threshold $\bar{p}$ corresponds to the smallest PCC value warranting that no false positive links are included. Finally, on average, for threshold values greater than $\bar{p}$, the derivative of HIM distance is larger than before $\bar{p}$, while the false negative rate remains under 0.8.

## Conclusions

We have proposed a simple a priori, theoretical and non-parametric method for the selection of a hard threshold for the construction of correlation networks. This model is based on the requirements of filtering random data due to noise and reducing the number of false positives, and it is implemented by means of analytic properties of PCC. This new approach can be especially useful where there is a small sample size and when the task requires minimising the number of false positive links, probably the most common situation in profiling studies in functional genomics. Finally, when the number of samples increases, coupling this method with soft thresholding approaches, can help recovering false negative links neglected by overly strict thresholds.

## Supporting Information

S1 Text Supplementary Text and Figures. (PDF)
