# Supplementary material for: A Null Model for Pearson Coexpression Networks
Source: PLoS One. 2015 Jun 1;10(6):e0128115. doi: 10.1371/journal.pone.0128115 (PMC4452659; doi:10.1371/journal.pone.0128115)
Supplement: S1 Text — (PDF) [file pone.0128115.s001.pdf]

A null model for Pearson coexpression networks Supplementary Material – S1 Text

Andrea Gobbi, Giuseppe Jurman*
Fondazione Bruno Kessler, Trento, Italy
* E-mail: jurman@fbk.eu

Text A The Pearson Correlation Coefficient 1

Text B A 3-dimensional example of Proposition S1 5

Text C Main moments of the Pearson correlation 6

Text D The restricted secure threshold $\tilde{p}_k$ 6

Text E Functional relations undetected by correlation networks 7

Text A The Pearson Correlation Coefficient

Let $x, y \in \mathbb{R}^n$ with $n \geq 3$. Then the Pearson Correlation Coefficient (PCC) ρ between x and y is defined as:

$$\rho(x,y) = \frac{\sum_{i=1}^{n}(x_i - \overline{x})(y_i - \overline{y})}{\sqrt{\sum_{i=1}^{n}(x_i - \overline{x})^2}\sqrt{\sum_{i=1}^{n}(y_i - \overline{y})^2}} ,$$

where $\overline{x}, \overline{y}$ denote the arithmetic means $\frac{1}{n}\sum_{i=1}^{n} x_i$ and $\frac{1}{n}\sum_{i=1}^{n} y_i$ respectively.

Define two new random variables $\tilde{x}$ and $\tilde{y}$ as follows:

$$\tilde{x} = \frac{x - \overline{x}}{\sigma_x\sqrt{n-1}}, \qquad \text{and} \qquad \tilde{y} = \frac{y - \overline{y}}{\sigma_y\sqrt{n-1}} , \tag{S1}$$

where σ_x and σ_y are the standard deviations of x and y.

From the definition, the following identities immediately descend:

$$\begin{aligned} \sum_{i=1}^{n} \tilde{x}_i &= 0 = \sum_{i=1}^{n} \tilde{y}_i , \\ \sum_{i=1}^{n} \tilde{x}_i^2 &= 1 = \sum_{i=1}^{n} \tilde{y}_i^2 , \\ \rho(x,y) = \rho(\tilde{x},\tilde{y}) &= \sum_{i=1}^{n} \tilde{x}\tilde{y} . \end{aligned} \tag{S2}$$

Since $\tilde{x}$ (and $\tilde{y}$) lies on the unit sphere because $\|\tilde{x}\| = 1$, Equation (S2) yields that

$$\begin{aligned}\sum_{i=1}^{n} \tilde{x}_i &= \sum_{i=1}^{n} \frac{x_i - \overline{x}}{\sigma_x \sqrt{n-1}} \\ &= \frac{1}{\sigma_x \sqrt{n-1}} \sum_{i=1}^{n} (x_i - \overline{x}) \\ &= \frac{1}{\sigma_x \sqrt{n-1}} \left[\left(\sum_{i=1}^{n} x_i \right) - n\overline{x} \right] \\ &= \frac{1}{\sigma_x \sqrt{n-1}} (n\overline{x} - n\overline{x}) \\ &= 0 \ ,\end{aligned}$$

and the same holds for $\tilde{y}$, too.

Thus we can rephrase Equation (S2) as follows:

Proposition S1. *Let $x, y, \tilde{x}, \tilde{y}$ be as in Equation (S1). Then $\tilde{x}, \tilde{y} \in S_{n-1} \cap \mathcal{H} \simeq S_{n-2}$, where $\mathcal{H}$ is the vectorial hyperplane defined as $\sum_{i=1}^{n} w_i = 0$ and w_i are the coordinates of $\mathbb{R}^n$.*

An example for $n = 3$ of the situation described in Proposition S1 is shown in Section Text B.

To prove now Equation (2), we first combine Equation (S1) and Equation (S2) to obtain that

$$\rho(x, y) = \rho(\tilde{x}, \tilde{y}) = \tilde{x}\tilde{y} = \cos \beta \ , \tag{S3}$$

where β is the angle between the two vectors $\tilde{x}$ and $\tilde{y}$. Equation (S3) and Proposition S1 yield that $P(|\rho(x, y)| > p)$ is the proportion between the area of the spherical cap in $n - 2$ dimensions included within an angle β from x and the whole surface of the $(n - 2)$-dimensional sphere [1]. A compact formula for the area $A^{\text{cap}}_{n-1}(r)$ of a $(n - 2)$-dim spherical cap is given in [2] as:

$$A^{\text{cap}}_{n-1}(r) = \frac{2\pi^{(n-2)/2}}{\Gamma\left(\frac{n-2}{2}\right)} r^{n-2} \int_0^{\beta} \sin^{n-3}(\vartheta) \mathrm{d}\vartheta \ ,$$

and, since the area of the whole surface is

$$S_{n-2}(r) = \frac{2\pi^{(n-1)/2}}{\Gamma\left(\frac{n-1}{2}\right)} r^{n-2} \ ,$$

the thesis follows from the position $r = 1$.

In Proposition S1 the transformed vectors are assumed to be uniformly distributed on the spherical surface. This assumption holds in the case of a normal distribution, but it does not hold in general. However, in the following paragraph we show that it is a good approximation, since x and y are independent. In fact, Equation (2) can be generalised to other distributions [3–6], when data skewness can be bounded [1].

Let $G^{\delta}(n, p)$ be an empirical distribution generated by k couples of two vectors $x, y \in \mathbb{R}^n$ sampled according to a given distribution function δ. Let then

$$E_t(F, G^{\delta}) = \sqrt[t]{\int_0^1 |F(n, p) - G^{\delta}(n, p)|^t \mathrm{d}p}$$

be the t-error function evaluating the difference between the theoretical distribution $F(n,p)$ and the empirical distribution $G^\delta(n,p)$. Hereafter we report the results of the simulations for $k = 50000$ and $n = 8, 20, 100$, where δ is one of the following three distribution functions:

- $U(\min, \max)$, the uniform distribution in $[\min, \max]$;
- $N(\mu, \sigma)$, the normal distribution with mean μ and standard deviation σ;
- $L(\mu_{\log}, \sigma_{\log})$, the lognormal distribution with mean-log $\mu_{\log}$ and standard deviation-log $\sigma_{\log}$.

In particular, in Table S1 we list the values of $E_2(F, G^\delta)$ and in Figure S1 we display the curves of the Cumulative Distribution Functions (CDF) of $G^\delta(n,p)$ corresponding to the three functions δ, separately for the three different values of $n = 8, 20, 100$. The distribution parameters we used are $\min = 0, \max = 0, \mu = 0, \sigma = 1, \mu_{\log} = 2, \sigma_{\log} = 3$. Regardless of the value of n, the empirical distribution fits the exact formula Equation (2) when x and y are uniformly sampled, while it does not fit the same equation when the two vectors come from extremely skewed distributions such as the lognormal.

Table S1. Error function $E_2(F, G^\delta)$, for $n = 8, 20, 100$ and different distributions $\delta = U(0,1), N(0,1), L(2,3)$.

$G^\delta(8,p)$	$U(0,1)$	$N(0,1)$	$L(2,3)$
$U(0,1)$	0.001832	0.00137	0.021202
$N(0,1)$	0.001195	0.00142	0.001432
$L(2,3)$	0.022961	0.00139	0.080803

$G^\delta(20,p)$	$U(0,1)$	$N(0,1)$	$L(2,3)$
$U(0,1)$	0.0016851	0.0007752	0.0248819
$N(0,1)$	0.0008008	0.0014559	0.0008381
$L(2,3)$	0.0238804	0.0011422	0.1038271

$G^\delta(100,p)$	$U(0,1)$	$N(0,1)$	$L(2,3)$
$U(0,1)$	0.0006978	0.0008244	0.015630
$N(0,1)$	0.0009281	0.0007388	0.001441
$L(2,3)$	0.0159969	0.0014090	0.104998

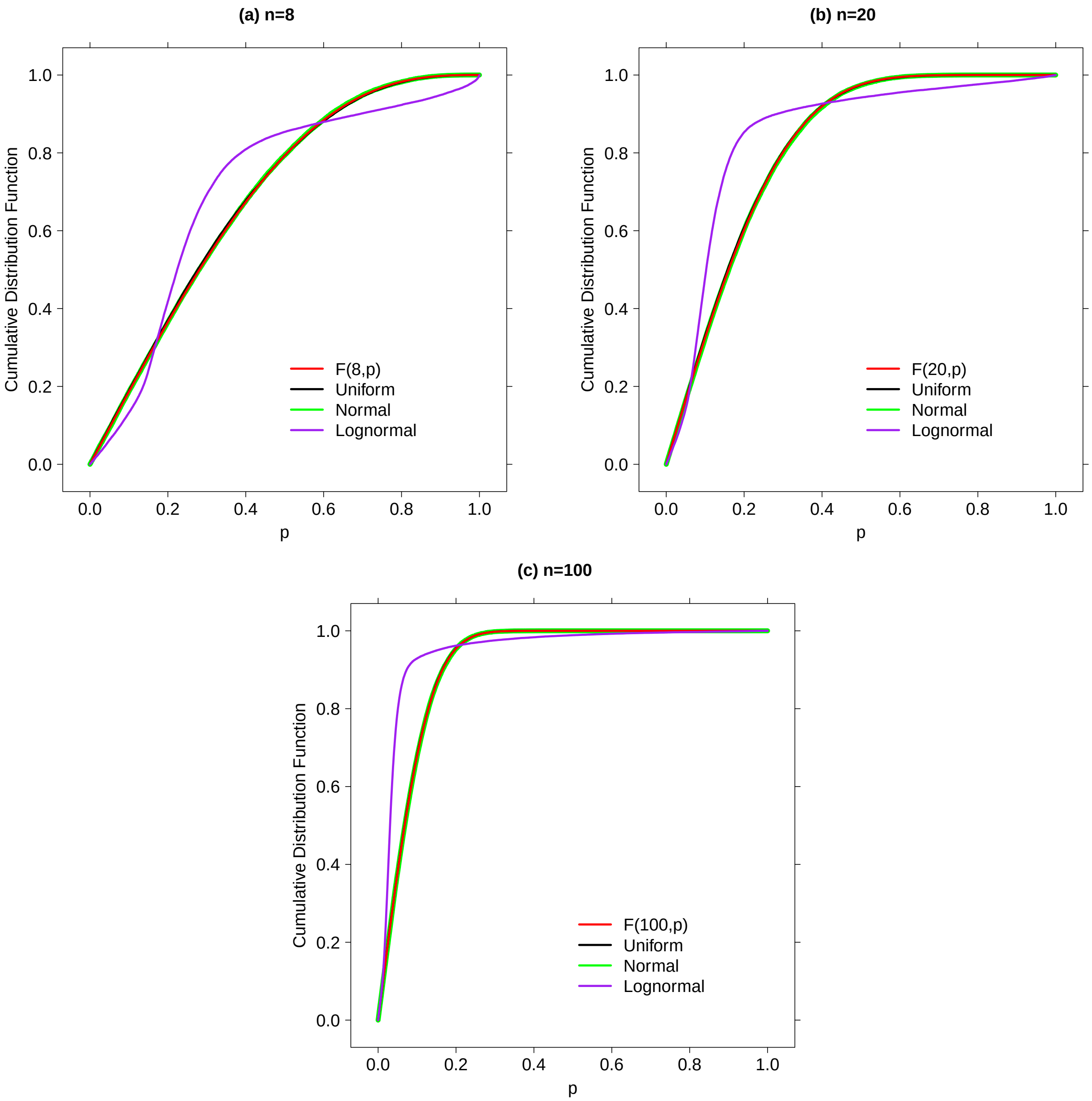

Figure S1. Cumulative Distribution Functions relative to the absolute value of Pearson correlation between 50,000 instances of pairs of n-dimensional vectors sampled from three different distributions Uniform ($\delta = U$, min $= 0$, max $= 1$, black line), Normal ($\delta = N$, $\mu = 0$, $\sigma = 10^4$, green line) and LogNormal ($\delta = L$, $\mu_{\log} = 2$, $\sigma_{\log} = 3$, purple line), compared with the theoretical curve $F(n,p)$ (red line), for the three cases $n = 8$ (a), $n = 20$ (b) and $n = 100$ (c). In all cases, the red curve of $F(n,p)$ and the black curve for the uniform distribution are almost coincident.

Text B A 3-dimensional example of Proposition S1

Consider a dataset $\mathcal{Y}$ consisting of $n = 3$ samples described by $m = 100$ genes. Then $\mathcal{Y}$ can be represented by 100 points in $[0, 1]^3 \subset \mathbb{R}^3$ as shown in Fig. S2(a). The new variables are built through a two-stages procedure applied to each gene. First the mean is subtracted, so the transformed dataset lies on the hyperplane $\mathcal{H}$ described in Proposition S1 as displayed in Fig. S2(b,c). Finally. each gene is normalized to unitary variance, and the resulting dataset lies on $S_{n-1} \cap \mathcal{H}$, which is the circumference in Fig. S2(d).

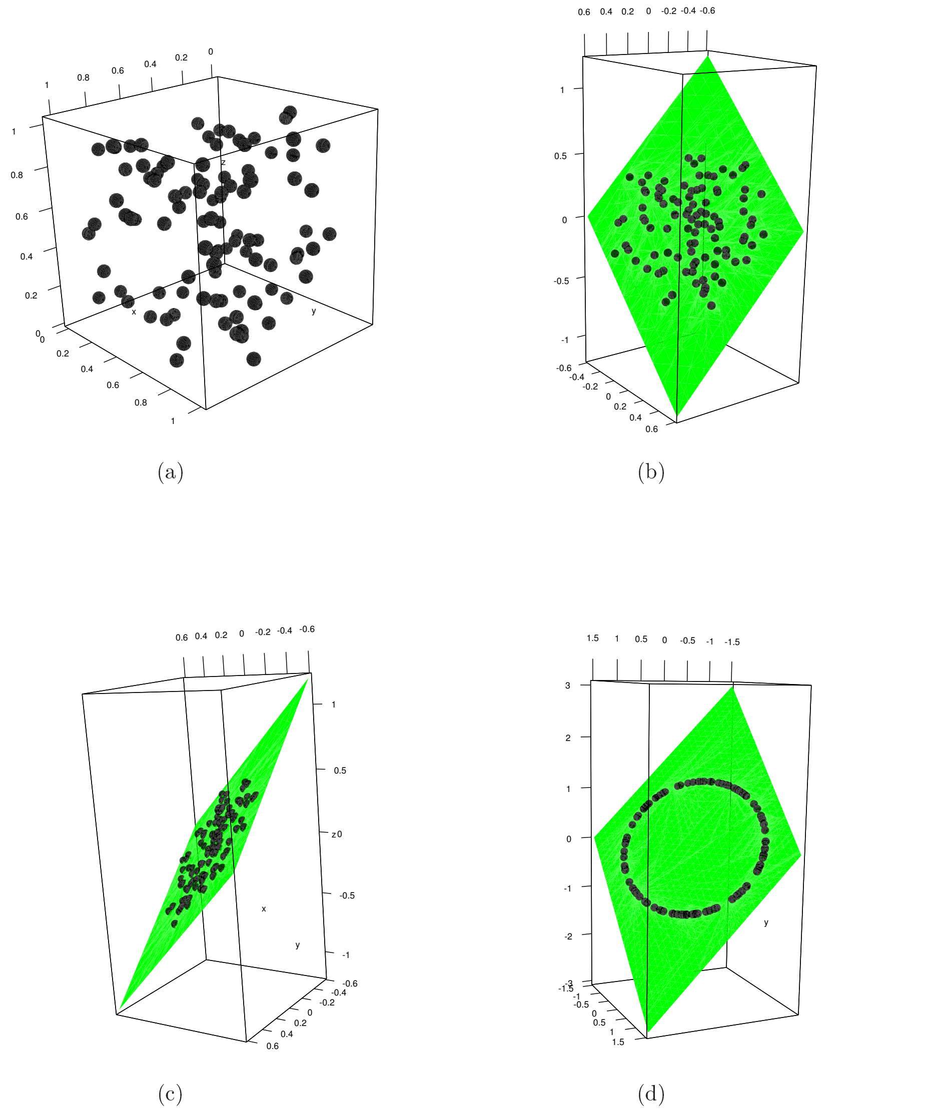

Figure S2. Transformation of the initial dataset preserving the Pearson correlation: (a) Original dataset (b,c) Mean substraction (d) Variance normalization. In green the hyperplane $\mathcal{H}$.

Text C Main moments of the Pearson correlation

Finally, we conclude deriving the mean and the variance of the function $|\rho|$ starting from Equation (2). The density function $f(n,p)$ can be computed as

$$f(n,p) = \frac{2}{\sqrt{\pi}} \frac{\Gamma\left(\frac{n-1}{2}\right)}{\Gamma\left(\frac{n-2}{2}\right)} (1-p^2)^{\frac{n-4}{2}} \ .$$

Using the above expression for $f(n,p)$, the two moments follow straightforwardly:

$$\mathbb{E}(|\rho|, n) = \int_0^1 p f(n,p) \mathrm{d}p = \frac{2}{\sqrt{\pi}} \frac{\Gamma\left(\frac{n-1}{2}\right)}{(n-2)\Gamma\left(\frac{n-2}{2}\right)}$$

$$\mathrm{Var}(|\rho|, n) = \int_0^1 p^2 f(n,p) \mathrm{d}p - \mathbb{E}^2(n,p) = \frac{1}{n-1} - \frac{4\Gamma^2\left(\frac{n-1}{2}\right)}{\pi(n-2)^2\Gamma^2\left(\frac{n-2}{2}\right)} \ .$$

Text D The restricted secure threshold $\tilde{p}_k$

We list here the analogue of Table 1 of Main Text for the restricted secure threshold

$$\tilde{p}_k = \min_{p \in (0,1]} \left\{ F(n,p)\frac{m(m-1)}{2} + k\sqrt{(1-F(n,p))F(n,p)\frac{m(m-1)}{2}} < 1 \right\} \ ,$$

for $k = 2$ and $k = 5$.

Table S2. A subset of values of the secure threshold $\tilde{p}_2$ for different number of samples m and genes n.

n \ m	100	500	1000	2000	10000	50000	100000
8	0.97584	0.99179	0.99484	0.99675	0.99889	0.99962	0.99977
15	0.86282	0.91826	0.93437	0.94723	0.96810	0.98065	0.98439
20	0.78966	0.85726	0.87876	0.89686	0.92883	0.95068	0.95784
30	0.68082	0.75573	0.78151	0.80425	0.84759	0.88074	0.89256
50	0.55034	0.62269	0.64902	0.67302	0.72135	0.76137	0.77651
75	0.45887	0.52436	0.54881	0.57145	0.61820	0.65834	0.67394
100	0.40153	0.46116	0.48369	0.50471	0.54865	0.58703	0.60214

Table S3. A subset of values of the secure threshold $\tilde{p}_5$ for different number of samples m and genes n.

n \ m	100	500	1000	2000	10000	50000	100000
8	0.98553	0.99508	0.99691	0.99805	0.99934	0.99978	0.99986
15	0.89287	0.93585	0.94842	0.95849	0.97486	0.98474	0.98768
20	0.82530	0.88080	0.89858	0.91361	0.94025	0.95853	0.96454
30	0.71934	0.78401	0.80647	0.82636	0.86445	0.89373	0.90420
50	0.58686	0.65162	0.67541	0.69720	0.74130	0.77803	0.79198
75	0.49164	0.55125	0.57373	0.59463	0.63803	0.67552	0.69015
100	0.43124	0.48595	0.50683	0.52640	0.56752	0.60368	0.61796

Text E Functional relations undetected by correlation networks

Correlation networks, like other univariate methods, are unable to capture relations between genes when the independence hypothesis does not hold. In these situations, quite common throughout -omics studies when the number of genes are much larger of the number of samples, the correlation values (*e.g.*, PCC) between expressions result negligible or small even when the corresponding genes are (functionally) related.

Hereafter we show three cases demonstrating the aforementioned behaviour.

1. **Toy model** Consider a simple system with four genes g_1, g_2, g_3, g_t, where the expression of gene g_t depends on the expression of $\{g_i\}_{i=1,2,3}$ according to the linear rule $g_t = g_1 + \frac{1}{2}g_2 + g_3$. Moreover, suppose that the expression of g_1, g_2 and g_3 is respectively uniformly, normally and gamma distributed, *i.e.* $g_1 \in U(0,1)$, $g_2 \in N(1,0)$ and $g_3 \in \Gamma(a = 0.1, s = 1)$, where the density of the gamma distribution is given by $f(x) = 1/(s^a\Gamma(a))x^{a-1}e^{-\frac{x}{s}}$. Finally, randomly extract the expression of g_1, g_2, g_3 on 100 samples, compute PCC(g_i,g_t) and repeat the experiment 10000 times. Although, by definition, g_t is strongly functionally related to g_1, g_2 and g_3, the corresponding average PCC are quite low, namely

	μ	σ
PCC(g_1,g_t)	0.70	0.12
PCC(g_2,g_t)	0.59	0.15
PCC(g_3,g_t)	0.28	0.27

   and thus the links between g_t–g_2 and g_t–g_3 are likely to be not detected in coexpression networks.

2. **ODE model** A similar situation occurs when the dynamics along time of the gene expressions is (more realistically) driven by a system of ordinary differential equations. Consider for instance the toy model on three genes g_1, g_2 and g_3 described in [7]: g_1 is repressed by g_3, g_2 is activated by g_1 and g_3 is activated by both g_1 and g_2.

   $$\begin{aligned}\dot{g_1} &= k_{1,s}\frac{1}{1+k_{1,3}g_3} - k_{1,d}g_1\\ \dot{g_2} &= k_{2,s}\frac{k_{2,1}g_1}{1+k_{2,1}g_1} - k_{2,d}g_2\\ \dot{g_3} &= k_{3,s}\frac{k_{3,1}g_1\cdot k_{3,2}g_2}{(1+k_{3,1}g_1)(1+k_{3,2}g_2)} - k_{3,d}g_3\ ,\end{aligned}$$

   where the reaction rate constants are set as follows: $k_{1,s} = 2$, $k_{2,s} = 2$, $k_{3,s} = 15$, $k_{1,d} = k_{2,d} = k_{3,d} = 1$, $k_{2,1} = k_{3,1} = 1$, $k_{3,2} = 0.01$, $k_{1,3} = 100$. Setting to zero all the initial conditions and solving the above ODE system for the time interval $t \in [0,5]$ and time step $\delta t = 0.01$ we obtain three time series on 501 points corresponding to the dynamics of the gene expressions for g_1, g_2 and g_3, whose curves are plotted in Fig. S3. Again, despite the strong functional relation among g_1, g_2 and g_3, some of the corresponding PCC values are quite small, namely PCC(g_1,g_2)=0.085 and PCC(g_1,g_3)=-0.288, while PCC(g_2,g_3)=0.927 for the only link which is likely to be inferred by the coexpression approach.

3. **A promoteromic example** The third example comes from the mammalian promoterome atlas of the FANTOM5 project [8]. Consider the time course on 16

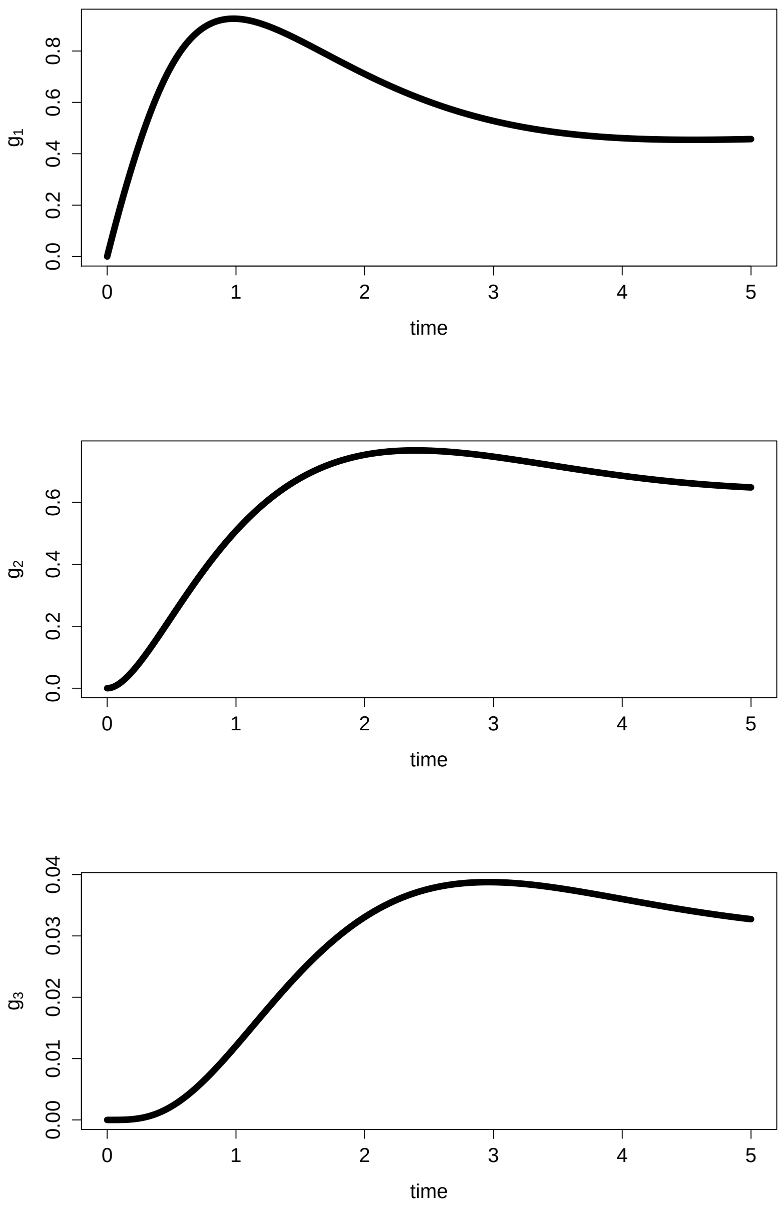

Figure S3. Example 2 Time series of the expression of genes g_1, g_2 and g_3 for the ODE synthetic model.

time points (0-480 minutes) of the expression of the three genes FOS, JUN and FOSL1, as shown in Fig. S4. Gene FOSL1 is regulated by both FOS and JUN: nonetheless, correlation between the target and the regulatory genes is negligible, namely PCC(FOSL1,FOS)=-0.22 and PCC(FOSL,JUN)=0.17, so that both links FOSL1–FOS and FOSL1–JUN are marked as non significant by a coexpression network approach.

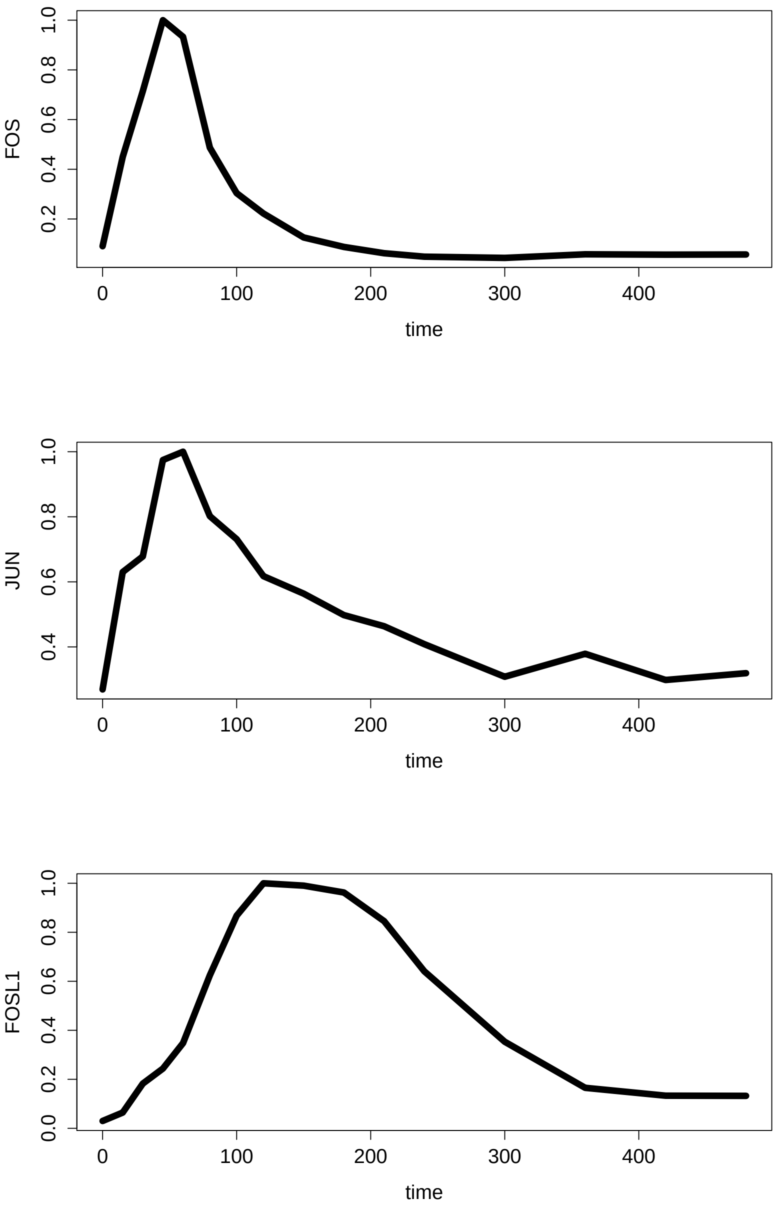

Figure S4. Example 3 Time series of the expression of genes FOS, JUN and FOSL1 for the FANTOM5 mammalian promoterome data.

References

1. Kendall M, Stuart A (1977) The Advanced Theory of Statistics: Distribution theory. Griffin, 472 pp.

2. Li S (2011) Concise Formulas for the Area and Volume of a Hyperspherical Cap. Asian Journal of Mathematics & Statistics 4: 66–70.

3. Gayen A (1951) The Frequency Distribution of the Product-Moment Correlation

Coefficient in Random Samples of any Size Drawn from Non-Normal Universes. Biometrika 38: 219–247.

4. Haldane J (1949) A note on non-normal correlation. Biometrika 36: 467–468.
5. Hey G (1938) A new method for experimental sampling illustrated in certain non-normal populations. Biometrika 30: 68–80.
6. Kowalski C (1972) On the Effects of Non-Normality on the Distribution of the Sample Product-Moment Correlation Coefficient. Journal of the Royal Statistical Society Series C (Applied Statistics) 21: 1–12.
7. Karlebach G, Shamir R (2008) Modelling and analysis of gene regulatory networks. Nature Reviews Molecular Cell Biology 9: 770–780.
8. The FANTOM Consortium and the RIKEN PMI and CLST (DGT) (2014) A promoter-level mammalian expression atlas. Nature 507: 462–470.
